# Localized iron accumulation precedes nucleation and growth of magnetite crystals in magnetotactic bacteria

**DOI:** 10.1038/s41598-017-08994-9

**Published:** 2017-08-15

**Authors:** Jacques Werckmann, Jefferson Cypriano, Christopher T. Lefèvre, Kassiogé Dembelé, Ovidiu Ersen, Dennis A. Bazylinski, Ulysses Lins, Marcos Farina

**Affiliations:** 1 Instituto de Ciências Biomédicas, Universidade Federal do Rio de Janeiro, 21941-902 Rio de Janeiro, Brazil; 2 Instituto de Microbiologia, Universidade Federal do Rio de Janeiro, 21941-902 Rio de Janeiro, Brazil; 3 CNRS/CEA/Aix-Marseille Université, UMR7265 Institut de biosciences et biotechnologies, Laboratoire de Bioénergétique Cellulaire, 13108 Saint Paul lez Durance, France; 4 Institut de physique et chimie des matériaux de Strasbourg (IPCMS) UMR 7504 CNRS 23 rue du Lœss, BP 43 67034 Strasbourg Cedex 2, France; 5 School of Life Sciences, University of Nevada at Las Vegas, Las Vegas, Nevada 89154-4004 USA

## Abstract

Many magnetotactic bacteria (MTB) biomineralize magnetite crystals that nucleate and grow inside intracellular membranous vesicles that originate from invaginations of the cytoplasmic membrane. The crystals together with their surrounding membranes are referred to magnetosomes. Magnetosome magnetite crystals nucleate and grow using iron transported inside the vesicle by specific proteins. Here we address the question: can iron transported inside MTB for the production of magnetite crystals be spatially mapped using electron microscopy? Cultured and uncultured MTB from brackish and freshwater lagoons were studied using analytical transmission electron microscopy in an attempt to answer this question. Scanning transmission electron microscopy was used at sub-nanometric resolution to determine the distribution of elements by implementing high sensitivity energy dispersive X-ray (EDS) mapping and electron energy loss spectroscopy (EELS). EDS mapping showed that magnetosomes are enmeshed in a magnetosomal matrix in which iron accumulates close to the magnetosome forming a continuous layer visually appearing as a corona. EELS, obtained at high spatial resolution, confirmed that iron was present close to and inside the lipid bilayer magnetosome membrane. This study provides important clues to magnetite formation in MTB through the discovery of a mechanism where iron ions accumulate prior to magnetite biomineralization.

## Introduction

Over 60 different minerals are known to be produced by organisms in a process called biomineralization^1^. In biomineralization, organisms passively or actively, but selectively, accumulate chemical elements from the environment and transform them into mineral structures inside or outside the cell. Biomineralization processes play crucial roles in ecosystems as many of these organisms participate in the geochemical cycles of major elements necessary to life^2^. In the prokaryotes, one remarkable example of biomineralization is the synthesis of chains of nano-sized, membrane-bounded, iron-rich magnetic mineral crystals called magnetosomes by magnetotactic bacteria (MTB). These intracellular chains of organelles, either composed of magnetite (Fe_3_O_4_) or greigite (Fe_3_S_4_), impart to the cell a sufficiently large magnetic moment to allow for the passive alignment of the bacteria in the Earth’s geomagnetic field^3^. This passive alignment associated with active swimming modulated by aerotaxis is responsible for the localization and positioning of MTB at an optimal position, the oxic-anoxic transition zone, in sediments and water where they thrive^4^.

In MTB, the magnetosome biomineralization process is under strict biochemical and genetic control^5–10^. Specific genes/proteins are involved in the biomineralization of the magnetosome crystals, production of the enveloping membrane, in the transport of Fe from outside the cell to the magnetosome vesicle, and the organization of the magnetosomes in chains^11^. The genes involved in magnetosome formation are called *mam* (magnetosome membrane) or *mms* (magnetic particle-membrane specific) genes and are usually clustered in a relatively, large, single chromosomal region in the genome. In several species of MTB, this region has been referred to as a genomic magnetosome island (MAI)^12, 13^. The magnetosome island composes about 100 kb (~2% of the genome) in *Magnetospirillum gryphiswaldense* strain MSR-1. To synthesize magnetosomes, MTB must take up the elements necessary for mineral formation from their surroundings. In the case of Fe_3_O_4_, Fe and O must be directed into the magnetosome vesicle. Fe can be taken up as either reduced or oxidized Fe compounds^14^, whereas O in Fe_3_O_4_ originates from water in *Magnetospirillum* and *Magnetovibrio* strains^15^. Different precursors have been proposed for Fe_3_O_4_ formation after Fe is transported across the outer membrane and enters the cell. The putative precursors include ferrihydrite, hematite, or high-spin reduced Fe complexes^16–19^. Recently, a mechanism involving phase transformations from disordered phosphate-rich Fe hydroxide into Fe_3_O_4_ via oxidized Fe oxyhydroxide intermediates was proposed for magnetite magnetosome formation^20^.

Determining the precise spatial distribution of different elements in cells of uncultivated and cultivated MTB may provide important information for understanding the biomineralization processes during magnetosome formation and the potential biogeochemical roles for MTB in natural environments. Although electron microscopy has been used extensively in structural and magnetic microstructure imaging of cells of MTB and their magnetosomes^21, 22^, high-resolution localization of Fe and other elements with state of the art analytical energy dispersive X-ray spectroscopy (EDS) and electron energy loss spectroscopy (EELS) in the cell and/or magnetosomes has not been performed. Here, we introduce a new level of sub-nanometric chemical characterization of MTB using a combination of analytical scanning transmission electron microscopy (ASTEM), EDS, and EELS with an electron beam spot size less than 0.2 nm. The purpose of the study was to determine whether these techniques could help to elucidate the chemical/biochemical pathway of Fe_3_O_4_ biomineralization in MTB. New and important results concerning the process of Fe_3_O_4_ biomineralization were obtained from 5 types of MTB. These findings include: (1) Fe_3_O_4_ magnetosomes are surrounded by a matrix that seems to sequester significant amounts of Fe ions; and (2) Fe ions accumulate around all faces outside the magnetosome crystal and inside the lipid bilayer membrane of magnetosomes before their transfer to the forming crystal probably due to the actions of specific proteins. These findings together suggest that Fe migration and accumulation mechanisms precede nucleation and growth of Fe_3_O_4_ crystals in MTB.

## Results

Three types of preparation were used in this work to achieve Fe mapping of MTB at the nanoscale and sub-nanoscale: (a) isolated magnetosomes; (b) whole-mount unfixed cells; (c) ultra-thin sections of cryo-fixed and freeze-substituted cells.

**Figure 1:**
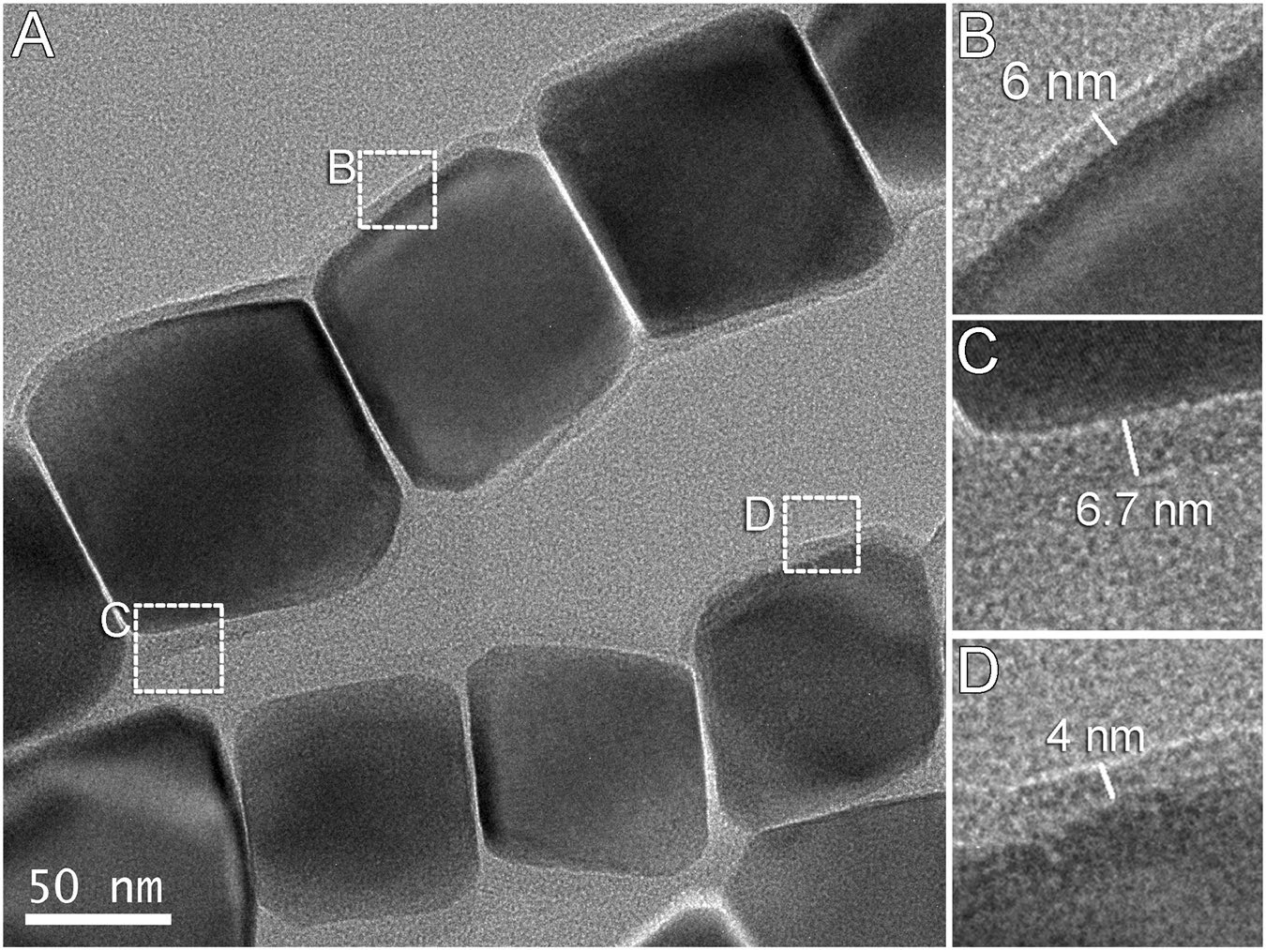
Transmission electron microscopy (TEM) images of purified Fe_3_O_4_ magnetosomes from *Magnetofaba australis* strain IT-1. (**A**) Linear chains of elongated cuboctahedral magnetosomes surrounded by what appears to be a continuous organic layer. (**B–D**) High magnification views of regions in A, showing the organic layer (the magnetosome membrane) of various thicknesses.

The magnetosome membrane is essential for magnetosome biomineralization and growth of Fe_3_O_4_ crystals inside the bacteria^3, 11^. Thus, imaging isolated magnetosomes from cultured bacteria and inside whole mounts of uncultured magnetotactic cocci was done with conventional transmission electron microscopy (CTEM), high resolution transmission electron microscopy (HRTEM), scanning transmission electron microscopy (STEM) and high angle annular dark-field (HAADF) to determine the best imaging conditions. The magnetosome membrane was clearly observed surrounding magnetosome Fe_3_O_4_ crystals in purified magnetosomes from *Magnetofaba australis* strain IT-1 (Fig. 1A) and in whole mounts of the various uncultured cocci. The images obtained in HRTEM mode are phase contrast images. Depending of the sample thickness, the defocus value for the objective lens introduces a bright line at each interface resulting from the Fresnel diffraction^23^ effect, which helps in detecting the surrounding membrane (see Supplementary Fig. S1). The membrane thickness was not uniform varying from 3 nm to 7 nm (Fig. 1B–D). In the space between two adjacent crystals in a chain, thickness is difficult to measure because of defocus, which introduces a bright contrast that blurs the edges of the crystals (Fig. 1A). The value is estimated at 1 nm. In STEM-HAADF images, the contrast is mass-thickness dependent^24^ and the presence of the membrane is highlighted by adjusting the luminosity-contrast ratio (Supplementary Fig. S2).

To obtain an overall view of MTB cells and to preserve their ultrastructure, high pressure freezing followed by freeze-substitution and uranyl acetate staining was performed. The ultrastructure of *Magnetofaba australis* strain IT-1 (see Supplementary Fig. S3) and the uncultivated freshwater cocci from Strasbourg, France (see Supplementary Fig. S3) showed that both types of cells contained a large quantity of empty vacuoles and large phosphate-rich granules. Magnetosomes appeared to be surrounded by a large quantity of organic matter visualized as a large amount of condensed or concentrated, electron-dense material.

Structural and chemical information at the sub-nanoscale were also obtained on bacterial cells and isolated magnetosomes which were directly deposited on holey carbon grids; in this case, the overall ultrastructure may be lost but magnetosomes and surrounding macromolecules are conserved.

### EDS of cells and magnetosomes

**Figure 2:**
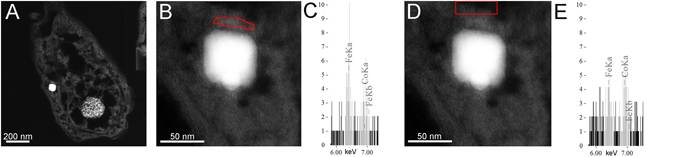
STEM-HAADF of thin-sections of a cryo-fixed cell from cultured *Magnetofaba australis* strain IT-1. (**A**) Low magnification image showing the cytoplasm and a Fe_3_O_4_ magnetosome. (**B** and **D**) show high magnification images of the Fe_3_O_4_ magnetosome. The selected regions in (**B** and **D)** are the masks used to extract the EDS spectra shown in (**C** and **E)**, respectively. By comparing the relative intensities of Fe and Co in both spectra, we conclude that iron is more concentrated in the region near the magnetosome (**C**) than in the cytoplasm (**E**). It can be seen that, compared to Co, the Fe signal decreases as the distance from the magnetosomes increases.

**Figure 3:**
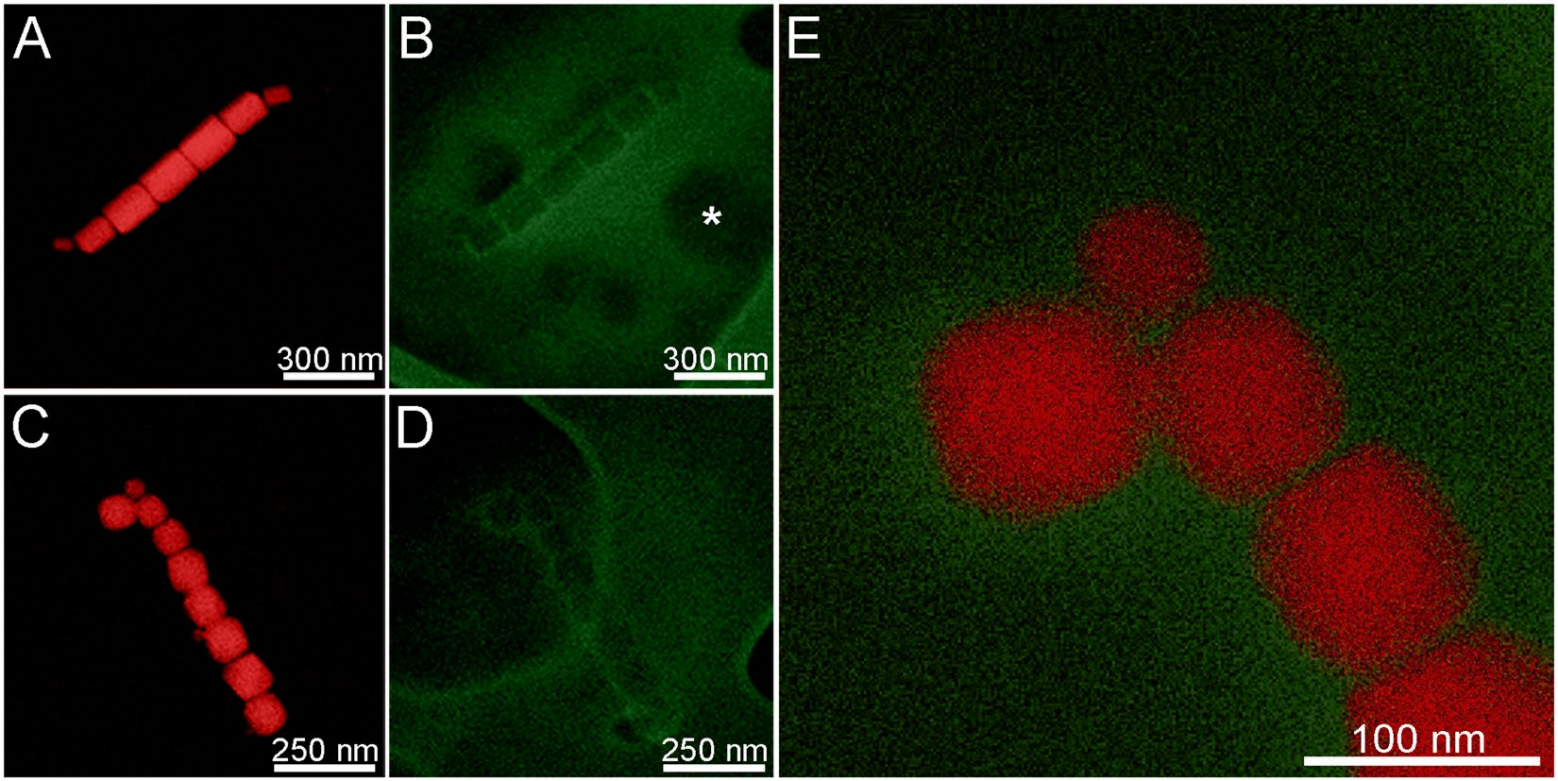
EDS maps of cells of uncultured coccoid MTB from the Mediterranean Sea (**A,B**) and cultured *Magnetofaba australis* strain IT-1 (**C,D**). (**A**) Fe map of the uncultured coccus. (**B**) Corresponding C map of area in (**A)**. (**C**) Fe map of the cultured *M. australis* strain IT-1 (**D**) Corresponding (**C**) map of area in (**C)**. (**E**) High magnification superposition elemental map of Fe (red) and C (green) of *M. australis* strain IT-1. The green region that surrounds the magnetosome in (**E**) corresponds to the magnetosomal matrix. White arrows in (**B** and **D)** highlight the contour of the hole in the formvar lacey, and asterisk the position of one granule. (Jeol ARM microscope, Beam intensity 1 nA).

Elemental composition of cells is described in supplementary Fig. S4. Analysis at high resolution showed a significant localization of Fe close to the magnetosome surface (Fig. 2). Using masks (Fig. 2), the presence of Fe is higher in the vicinity of magnetosomes (Fig. 2B and C) than at a larger distance from magnetosome (Fig. 2D and E). In air-dried MTB cells directly deposited on lacey carbon grids, microstructure and magnetosome chains were easily identified by EDS analysis (Fig. 3). Magnetosome chains were surrounded by a region of high C density although it is not possible to give an exact limit to this region, but it seems larger, up to 20 nm thick, in cells of both uncultured Mediterranean cocci and cultured *Magnetofaba australis* strain IT-1 (Fig. 3B and D). A high magnification superposing map of both Fe and C is shown in Fig. 3E. A carbonaceous “corona” surrounded the Fe_3_O_4_ crystals.

**Figure 4:**
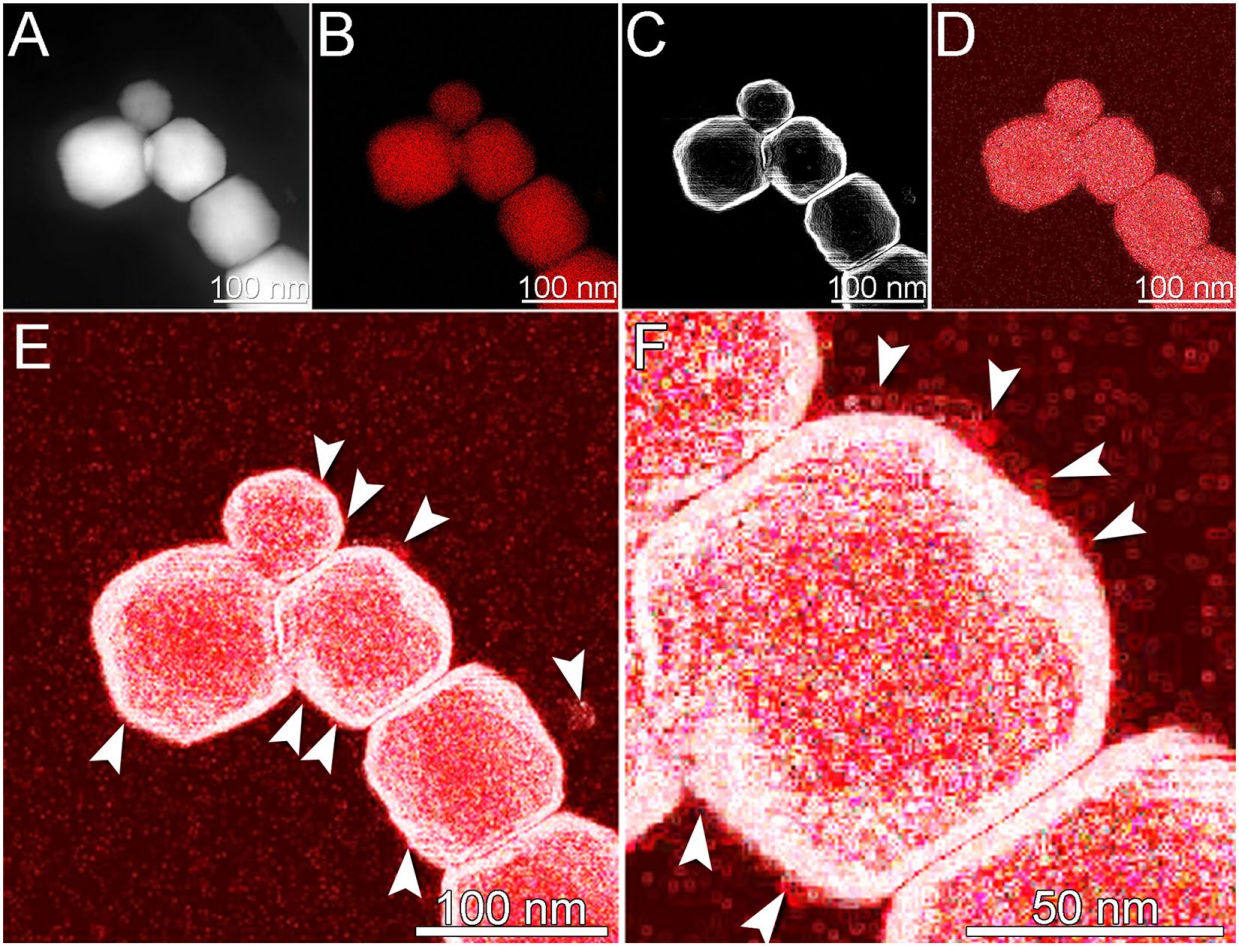
STEM images and EDS elemental maps of magnetosome chains from cells of cultured *Magnetofaba australis* strain IT-1. (**A**) HAADF image of a Fe_3_O_4_ magnetosome chain. (**B**) Fe map of the chain shown in (**A**). (**C**) Edge detection algorithm image of the STEM image shown in (**A**). (**D**) Edge detection algorithm image of the Fe map shown in (**C**). (**E**) Overlay image of the processed STEM (**C**) and the Fe map (**D**). (**F**) High magnification of the overlay image shown in (**E**). Arrows indicate regions of significant Fe accumulation around the magnetosome Fe_3_O_4_ crystal.

An edge detection algorithm was used to qualitatively highlight the presence of Fe outside the crystal. The algorithm was applied to STEM-HAADF images (Fig. 4A) which defined a contour corresponding to the limit of the crystal (Fig. 4C). The same algorithm was applied to the Fe map (Fig. 4B), modifying the contrast-luminosity ratio (Fig. 4D). The superposition of STEM-HAADF and Fe map images highlighted the presence of very low amounts of Fe outside the magnetosome Fe_3_O_4_ crystal in the form of aggregation of image pixels (Fig. 4E and F).

**Figure 5:**
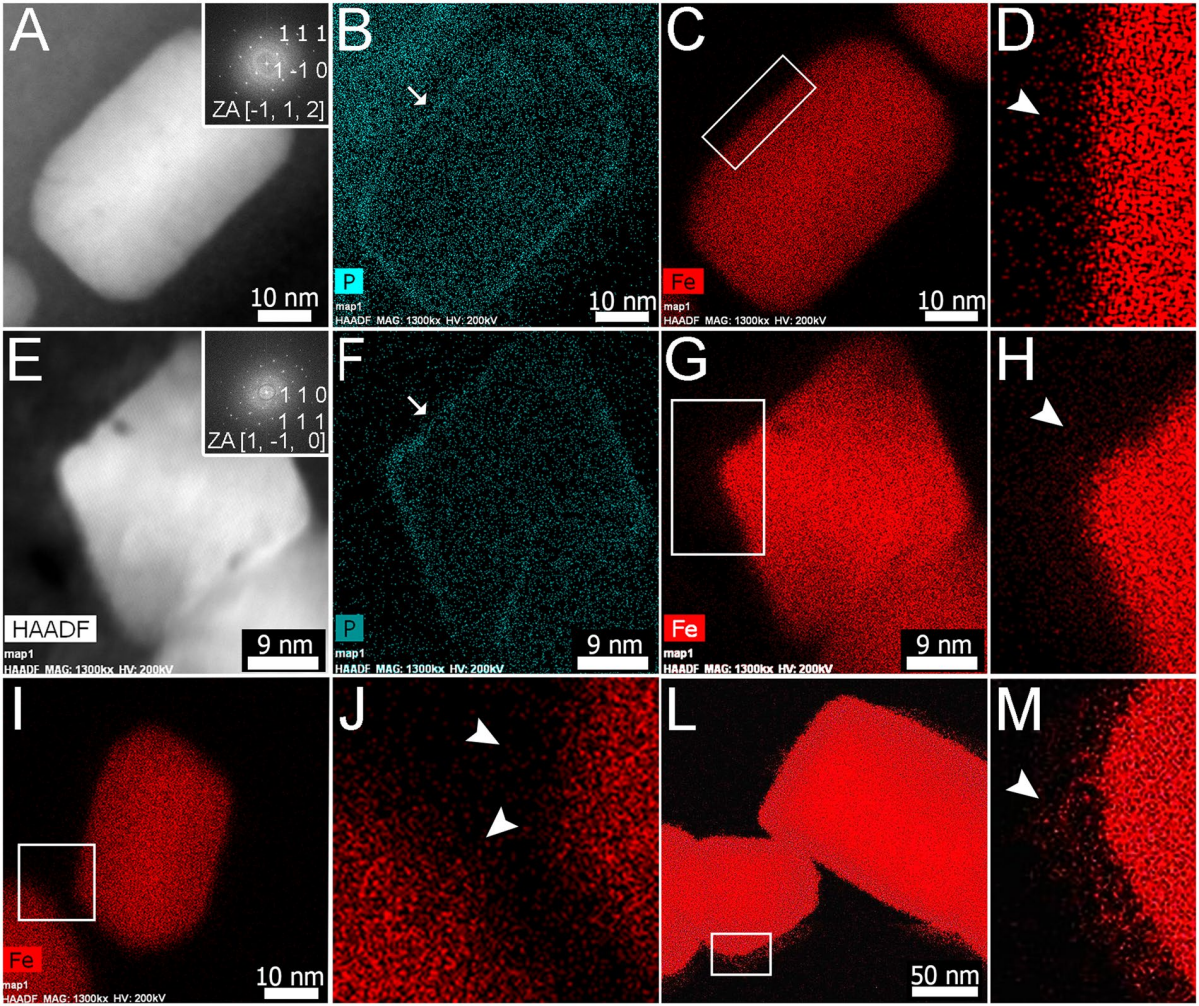
High resolution STEM imaging and EDS elemental maps of cells of cultured: *Magnetovibrio blakemorei* strain MV-1 cell (**A**–**D** and **I,J**), *Magnetofaba australis* strain IT-1 (**E**–**H**) and from uncultured coccoid MTB from the Mediterranean Sea (**L**,**M**). (**A**) STEM-HAADF image showing a prismatic Fe_3_O_4_ magnetosome observed edge-on. Inset shows fast Fourier transform (FFT) analysis of Fe_3_O_4_ with indexation. (**B**) P map of the region shown in (**A**). Arrow denotes the magnetosome membrane evidenced by the higher intensity of P due to the presence of phospholipids. (**C**) Fe map of the region shown in (**A**). (**D**) High magnification Fe map of the region shown in (**C**) showing the presence of Fe surrounding the magnetosome (at arrow). (**E–H**) same as (**A**) to (**D**) but for *Mf. australis* strain IT-1. (**I**) Fe map of Fe_3_O_4_ magnetosome from *Mv. blakemorei*. (**J**) Enlarged Fe map of the intermagnetosome region of cell of *Mv. blakemorei* inside (**I**). Note the diffuse Fe distribution surrounding magnetosomes (arrowheads). (**L**) Fe map of Fe_3_O_4_ magnetosome from a cell of the uncultured magnetotactic coccus from the Mediterranean Sea. (**M**) High magnification image of a region of the Fe map inside the rectangle in (**L**) showing Fe surrounding the magnetosome (arrowhead). (Beam intensity FEI Titan microscope 1.3 nA figures (**A**–**J**), Jeol ARM 1 nA figures (**L** and **M**).

A higher spatial element-specific resolution was obtained using aberration corrected STEM. Figure 5A and E show a whole-mount STEM image of cells of *Magnetovibrio blakemorei* strain MV-1 and *Magnetofaba australis* strain IT-1, and the coccus from the Mediterranean Sea, respectively. The lattice fringes of the crystals (Fig. 5A and E) and their corresponding FFT can be seen. Elemental mapping (Fig. 5B and F) shows a high density of blue pixel from the P signal enveloping each crystal corresponding to the magnetosome membrane. Elemental mapping of Fe (Fig. 5C and G) shows that the Fe signal appears as a corona around the crystal (Fig. 5D and H). Fe is found beyond the Fe_3_O_4_ crystal and magnetosome membrane reaching into the cytoplasm. By using the same edge algorithm described above, the superposition of HAADF images and Fe maps (see supplementary Fig. S5), shows that Fe completely surrounds the Fe_3_O_4_ crystal. The presence of the Fe corona in the cytoplasm was not generally observed in all cases. In some magnetosome chains in coccoid MTB, Fe was only detected inside the membrane (see supplementary Fig. S6). The presence or absence of the iron corona could indicate: (i) different steps of the biomineralization process or (ii) different Fe_3_O_4_ biomineralization pathways for different MTB strains.

### EELS analyses of cells and magnetosomes

**Figure 6:**
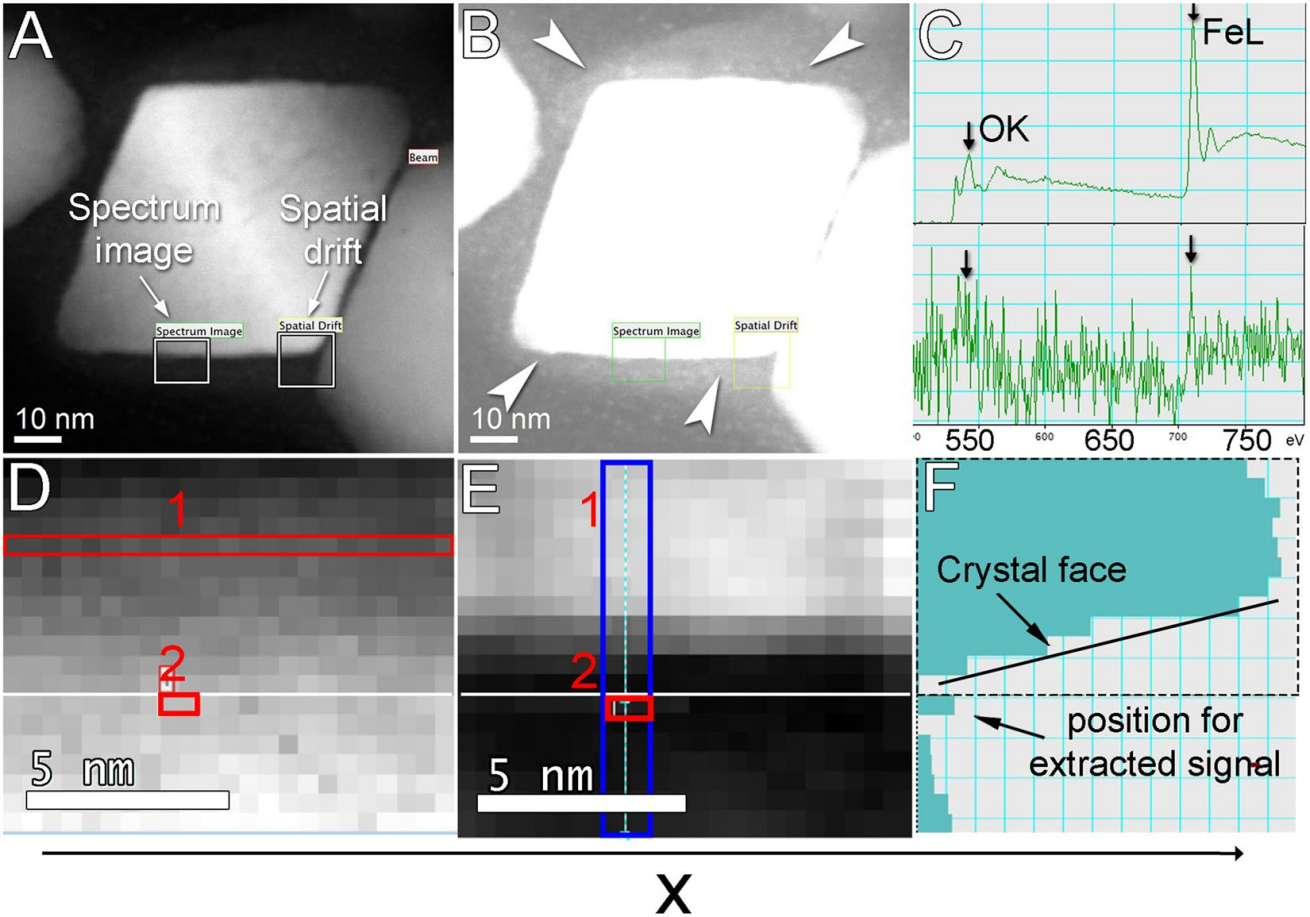
Electron energy loss spectroscopy (EELS) of an isolated Fe_3_O_4_ magnetosome from *Magnetofaba australis* strain IT-1 analyzed with spectral imaging at high spatial resolution (dwell time 2 s, beam intensity 0.038 nA camera length 2 cm, pixel size 0.5 nm). (**A**) STEM image of a magnetosome Fe_3_O_4_ crystal showing areas used for the analysis and drift correction (white rectangles). (**B**) Contrast stretching of the same image shown in (**A**) highlighting the surrounding membrane (white arrows). (**C**) EELS spectra obtained by the addition of spectra contained in each pixel of the same scanning line in (**D**); top: Spectrum corresponding to horizontally elongated rectangle (line 1) showing characteristic edges (arrows) for O (540 eV) and Fe (710 eV); bottom: Spectrum corresponding to line 2 obtained by the addition of 2 pixels along the line showing characteristic edges (arrows) for O (540 eV) and Fe (710 eV). (**D**) Spectrum obtained from the region shown in the square box in (**A**). Each pixel corresponds to one spectrum (pixel size 0.5 nm × 0.5 nm). (**E**) STEM-HAADF thickness map or profile map of the square region shown in (**A**). The intensity of each pixel is proportional of the sample thickness. (**F**) Projection profile of the addition of two pixels along the X axis of the HAADF intensity contained in the blue rectangle in (**E**). From bottom of (**C, D, E** and **F**), we assume that: (1) position 2 is outside the crystal (**D,E** and **F**), (2) in position 2 there is Fe (bottom of **C**) and thus Fe (other than Fe_3_O_4_) is inside the magnetosome membrane vesicle.

**Figure 7:**
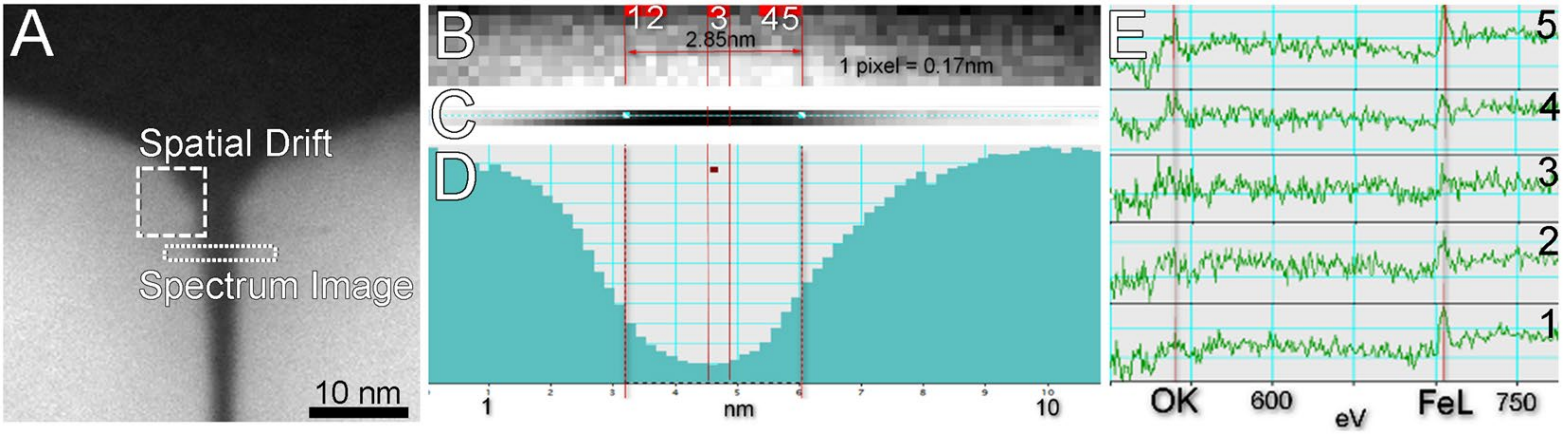
EELS of adjacent Fe_3_O_4_ magnetosomes in a cell of an uncultured freshwater coccoid MTB from a pond in Strasbourg France. (**A**) STEM HAADF image of two contiguous Fe_3_O_4_ magnetosomes in a chain. Spectrum image acquisition region and spatial drift (dwell time 2 s, camera length 2 cm, pixel size 0.17 nm). (**B**) Spectrum image obtained in the region highlighted in (**A**). Each acquired pixel can be assigned to an individual spectrum shown in figure (**E**). To enhance the signal to noise ratio, the signal from two pixels was added to give spectra in (**E**). (**C**) HAADF image or morphological map of the first line of STEM image acquired simultaneously as the spectrum image. (**D**) Morphological profile of the region between two magnetosomes. (**F**) EELS signals obtained from positions 1, 2, 3, 4, and 5 in spectrum image show in (**B**). Fe signals were found in all spectra.

EELS of magnetosomes was performed on purified magnetosomes and whole cells (Figs. 6 and 7, respectively). This method is well adapted for light element analysis and also for Fe giving a higher signal for the equivalent spectroscopic edge than EDS for the same microscope acquisition parameters. In the HAADF image (Fig. 6A) of extracted magnetosomes, the size and position of the acquired spectrum image (Fig. 6D) and its corresponding HAADF image (Fig. 6E) were defined as well as a spatial drift reference (Fig. 6A) for correcting the spatial drift during high resolution spectra acquisition with a dwell time of 2 sec. Contrast stretching was used to observe the surrounding magnetosome membrane which was about 10 nm thick (Fig. 6B). Figure 6C (top) shows, as a reference, a characteristic EELS spectrum acquired from a large part of the Fe_3_O_4_ crystal (Fig. 6D, region 1). Two characteristic edges were obtained for O and Fe, an O-K edge at 540 eV and a Fe-L edge at 710 eV. For sub-nanometric resolution a smaller beam diameter of 0.2 nm was used. The spectrum in Fig. 6C (bottom) was obtained by the addition of the intensity of two pixels (Fig. 6D, region 2) projected along the X direction which is the direction of the scan. The position for the extracted signal was correlated to the crystal face by the morphological profile (Fig. 6F). The small Fe signal obtained from position 2 is at one pixel (0.5 nm) far of the crystal face inside the surrounding lipid bilayer membrane (Fig. 6F).

To achieve higher spatial resolution, the analysis was conducted in space limited by two adjacent {111} crystal faces (Fig. 7A and B; FFT spectrum not shown). To limit the sequential drift correction effect (each 20 s), which reduces spatial precision, the first acquisition line was used. The morphological profile (Fig. 7D) obtained from the first line of the HAADF image (Fig. 7C) shows a flat minimum of about 0.8 nm between the crystals. At this minimum, the addition of two pixels of the spectral image (Fig. 7B) positions 2, 3 and 4 close to the minimum, gives a detectable Fe signal (Fig. 7E). In positions 1 and 5, the beam was probably in contact with the crystal. The analysis of cryofixed thin-sections of uncultivated magnetotactic cocci from Strasbourg, France, using a beam of 1 nm and a short dwell time of 0.1 s without the need of drift correction (see supplementary Fig. S7), showed the presence of Fe within the surrounding magnetosome membrane.

## Discussion

In all genomes of MTB, about thirty genes/proteins are known to be involved in Fe_3_O_4_ biomineralization in MTB^25^ with at least 10 thought to be essential for the synthesis of Fe_3_O_4_ magnetosome synthesis and magnetosome chain formation^11^. These proteins are involved in each of the steps thought to occur in Fe_3_O_4_ magnetosome biomineralization which include: magnetosome vesicle formation, Fe uptake and transport to the vesicle, nucleation of the crystal, and alignment of the magnetosomes into a chain^11^. In this study, we analyzed whole cells and extracted Fe_3_O_4_ magnetosomes from a number of cultured and uncultured MTB from natural environments using cryo-fixed, freeze-substituted samples and a combination of analytical techniques including ASTEM and high sensitivity EDS mapping and EELS for the purpose of elucidating the chemical/biochemical pathway of Fe_3_O_4_ biomineralization in MTB. All Fe_3_O_4_ magnetosome particles examined in this study were well developed crystals with characteristic faces of the cubic system as previously described^26, 27^.

Although phospholipid bilayer membranes are generally known to have a thickness of about 3 nm^28^, this value does not account for the lateral interactions among the membrane lipids and embedded proteins responsible for changes in the properties of a particular bilayer membrane, e.g., local thickness and curvature. These deformations result from hydrophobic interactions between membrane lipids and embedded proteins^29^. Magnetosome membrane thicknesses in the MTB studied here varied from 3 nm to 6.7 nm (Fig. 1B to D). In cryo-fixed samples stained with uranyl acetate, embedded in epoxy resin and analyzed in STEM mode, the magnetosome membrane could not be visually observed (Fig. 2A,B and Supplementary Fig. S3). However, using ASTEM, it was possible to estimate membrane thickness. As shown in supplementary Fig. S4, P is present in the cytoplasm of all MTB cells studied but is locally concentrated in a layer surrounding the Fe_3_O_4_ crystals, presumably corresponding to P in the phospholipid bilayer of the magnetosome membrane (Fig. 5B and F). From this P map, we estimate the membrane thickness to be equal or greater than 3 nm.

Very little is known regarding the specific spatial localization of magnetosome-associated proteins and the role of the magnetosomal matrix in magnetosome synthesis. Taoka *et al*.^30^ described four components in *Magnetospirillum magnetotacticum* strain MS-1 including: the Fe_3_O_4_ crystal, the magnetosome membrane, interparticle connections and a 50 nm thick magnetosomal matrix surrounding the magnetosome. Mam12 was exclusively located in the magnetosome membrane, while Mam22 (MamA) was a part of the magnetosomal matrix. Yamamoto *et al*.^31^ did not observe a magnetosome matrix by studying *Magnetospirillum magneticum* strain AMB-1 but showed with atomic force microscopy that a 7 nm thick lipid bilayer wrapped the Fe_3_O_4_ crystal which was surrounded by 4 nm thick layer of MamA. They suggested that MamA may be bridging the interaction of some magnetosome-associated proteins with the magnetosome membrane. The differences in these studies regarding the spatial localization of the MamA protein is probably a result of the methods used in the preparation of cells in each case^31^.

We observed a 20 nm thick magnetosomal matrix using EDS mapping obtained by X-ray C-K recording (Fig. 3) with Fe inside the membrane as shown by EDS and EELS, (Figs 2 and 6) and outside, probably in the MamA containing layer. Our data suggest that the magnetosomal matrix plays an essential role in directing or trapping Fe to and in that specific location. A region presenting lattice fringes surrounding Fe_3_O_4_ and Fe_3_S_4_ magnetosomes and aligned with the crystalline lattice fringes was previously described by Taylor and Barry^32^. Our observations using aberration-corrected HRTEM did not show similar regions.

A significant accumulation of Fe in the magnetosomal matrix and the presence of an Fe-rich layer might explain the rapid formation of Fe_3_O_4_ observed in time resolved studies, about 20 min after adding Fe to starved cultures of *Magnetospirillum gryphiswaldense* strain MSR-1^33^. However Schüler and Baeuerlein^33^ concluded that Fe_3_O_4_ magnetosome formation occurred without an accumulation of Fe ions at the magnetosome membrane. Our data indicate that, during crystal growth, Fe is concentrated in the magnetosomal matrix possibly allowing for a rapid accumulation of Fe around magnetosomes. Whole mounts observations of uncultured samples in this study appear to confirm this Fe accumulation model (Fig. 4F and E). The presence of a magnetosomal matrix, observed now in several MTB, naturally raises questions regarding of its physical and chemical structure and its role in the transfer of Fe through the magnetosome membrane for Fe_3_O_4_ formation. Our results in Figs 4, 5, 6 and 7, and supplementary Fig. S5 show that Fe ions surround all faces of the crystals studied. Because of the high spatial resolution (0.2 nm) of the analysis, Fe ions were also detected within the magnetosome membrane. Moreover, Fe was even detected by EELS between two successive crystals of a chain separated by a distance of less than 3 nm (Fig. 7C,D,E), confirming results obtained by EDS.

Analyses using the techniques described in this study might provide information regarding stages of crystal growth by comparing the density of Fe in each face. We hypothesize that the morphology of the crystallites is associated with Fe density in each face as it corresponds to the rate of growth of a specific face. This information might also indicate whether magnetosome crystals have reached a final stable uniform shape if some ratio between the Fe concentrations in the different faces is stable. This type of ratio analysis along a magnetosome might provide temporal information regarding Fe_3_O_4_ magnetosome synthesis by examining neighboring Fe_3_O_4_ crystals, while the comparison of Fe densities in each crystal face could provide details that result in specific crystal morphologies.

Two mechanisms have been proposed for Fe_3_O_4_ crystal formation in magnetosomes: (i) Fe is deposited quasi epitaxially on the Fe_3_O_4_ crystal face in formation deduced from *in vitro* Fe_3_O_4_ precipitation experiments in the presence of purified magnetosome-associated proteins which induce Fe_3_O_4_ growth and from their structural conformations analyses^9^. This ideal growth of Fe_3_O_4_ crystals does not explain the morphological diversity of Fe_3_O_4_ crystals that differ from cuboctahedral symmetry^34, 35^. (ii) Fe is precipitated in different chemical states before Fe_3_O_4_ formation without the involvement of specific proteins. A precursor of Fe_3_O_4_ exists in a mineralized state outside the magnetosome and three pathways were proposed for crystal formation: (a) soluble Fe is first oxidized into a ferrihydrite precursor, transported into the magnetosome, and further dehydrated and partially reduced to form Fe_3_O_4_^3, 36^, (b) Fe within a ferritin-like protein is co-precipitated, along with soluble Fe(II) ion, to form Fe_3_O_4_ crystallites at the cell membrane which then mature into fully formed Fe_3_O_4_ magnetosome crystals^18^, (c) transformation from disordered phosphate-rich Fe hydroxide into Fe_3_O_4_ *via* oxidized Fe oxyhydroxide intermediates^20^. We found no evidence at the nanoscale for a correlation between P and Fe concentrations in all species studied. Our results seem to be more consistent with the presence of a ferrihydrite or ferrihydrite-like precursor^41^ within the magnetosome matrix located around and within the phospholipid bilayer magnetosome membrane vesicle where the Fe_3_O_4_ crystal grows. We did not observe crystalline structures apart from magnetosome Fe_3_O_4_ in any of the analyses performed.

Based on the above considerations and our results, we postulate that the morphology of nanocrystals in the five MTB strains studied, and probably in all Fe_3_O_4_-producing MTB is induced by constraints imposed by the magnetosome membrane^37^, and by specific associated proteins that play roles in transferring ions across the membrane wherein the crystals are growing, allowing the formation of different types of crystal morphologies. The transfer of ions through the membrane is, at present, still unexplained, awaiting characterization at the atomic level.

## Materials and Methods

### Sampling, cultivation and purification of magnetosomes

Cultured and uncultured MTB cells collected from natural environments were examined by ASTEM, HRTEM and CTEM. These include uncultured magnetotactic cocci from the Itaipu lagoon (near Rio de Janeiro city, Brazil) from the freshwater Ill river, Doernel island Strasbourg, France, and coccoid bacteria from the Calanque of Mejean, Marseille, Mediterranean Sea as well as cells of the cultured MTB *Magnetofaba australis* strain IT-1 and *Magnetovibrio blakemorei* strain MV-1. For harvesting uncultivated MTB, water and sediment were collected using 1L plastic bottles that were stored at room temperature in the laboratory. Magnetic enrichment of MTB cells was done using a customized glass apparatus according to Lins *et al*.^38^, or using a properly aligned ordinary bar magnet attached to the plastic bottle. *Magnetofaba australis* strain IT-1 was grown in heterotrophic medium as previously described^39^. Cells of this strain were grown at 28 °C for approximately 15 days when a microaerophilic band of cells was obvious at the oxic-anoxic interface. *Magnetovibrio blakemorei* strain MV-1 was cultivated anaerobically in liquid medium with nitrous oxide (N_2_O) as the terminal electron acceptor and FeSO_4_•6H_2_O (100 μM) as the major source of Fe as previously described^40^. Cells of this strain were grown at 28 °C.

### Purification of magnetosomes

Cells were concentrated at the bottom of a polypropylene tube with a Nd-Fe-B magnet and lysed by resuspending them in a solution containing 1% sodium dodecyl sulfate (SDS) and 0.2 M NaOH at 60 °C for 15 min. Magnetosomes were magnetically concentrated for 1 h at 4 °C after which the supernatant was removed and then washed in buffer containing 10 mM HEPES and 200 mM NaCl (pH7.4) at 4 °C in a bath sonicator (Branson 2200) for 15 min. This washing process was repeated 5 to 10 times.

### High pressure freezing and cryo-substitution

For high pressure freezing and freeze substitution, cells of MTB were magnetically concentrated and high pressure frozen on a Leica HPM 100 high pressure freezing apparatus (Leica Microsystems, Bannockburn, IL, USA). Cells were then transferred to a fixative solution containing 2% uranyl acetate in anhydrous acetone in a freeze substitution Leica EM AFS2 apparatus (Leica Microsystems, Bannockburn, IL, USA). Samples were kept at −90 °C for 9 h, −35 °C for 4 h, and −20 °C for 2 h. The temperature was gradually increased to room temperature and cells were embedded and polymerized in PolyBed 812 (Polysciences, Warrington, PA, USA). Ultrathin sections were obtained using a Leica EM U6 ultramicrotome (Leica Microsystems, Bannockburn, IL, USA) and analyzed.

## Electron microscopy

Whole cells and purified magnetosomes were directly deposited on 400 mesh copper lacey carbon grids (Ted Pella, Inc., Redding, CA, USA). To disperse magnetosomes for more detailed analyses of individual magnetosomes rather than chains or clumps, suspensions of magnetosomes were sonicated prior to deposition on the grids. For X-ray mapping analytical imaging, an ASTEM JEOL JEM-ARM200F Cold FEG (JEOL Europe SAS., Croissy Sur Seine, France) equipped with a high sensitivity EDS setup obtained by 1 steradian large solid angle silicon drift detector (SDD) and a ASTEM FEI Titan 80–200 Cold FEG (Fei Co., Hillsboro, OR, USA) beam corrected for high resolution STEM equipped with four Bruker SDDs detectors (Madison, WI, USA) with an equivalent solid angle of collection as ASTEM JEOL, were used. A JEOL 2100 F ASTEM equipped with an EELS Tridiem detector (Gatan Inc., Pleasanton, CA, USA) was used to collect information for EELS spectra obtained from scanned lines adjacent and parallel to crystal faces. EDS image were processed using Image J free software program^42^.

## Electronic supplementary material

Supplementary info.
