# Supplementary material for: Localized iron accumulation precedes nucleation and growth of magnetite crystals in magnetotactic bacteria
Source: Sci Rep. 2017 Aug 15;7:8291. doi: 10.1038/s41598-017-08994-9 (PMC5557804; doi:10.1038/s41598-017-08994-9)
Supplement: Supplementary file 1 — Supplementary info. [file 41598_2017_8994_MOESM1_ESM.pdf]

Localized iron accumulation precedes nucleation and growth of magnetite crystals in magnetotactic bacteria

Jacques Werckmann[1,*], Jefferson Cypriano[2], Christopher T. Lefèvre[3], Kassiogé Dembelé[4], Ovidiu Ersen[4], Dennis A. Bazylinski[5], Ulysses Lins[2] and Marcos Farina[1]

1) Instituto de Ciências Biomédicas, Universidade Federal do Rio de Janeiro, 21941-902, Rio de Janeiro, Brazil

2) Instituto de Microbiologia, Universidade Federal do Rio de Janeiro, 21941-902, Rio de Janeiro, Brazil.

3) CNRS/CEA/Aix-Marseille Université, UMR7265 Institut de biosciences et biotechnologies, Laboratoire de Bioénergétique Cellulaire, 13108, Saint Paul lez Durance, France

4) Institut de physique et chimie des matériaux de Strasbourg (IPCMS) UMR 7504 CNRS 23 rue du Lœss - BP 43 67034 Strasbourg Cedex 2, France

5) School of Life Sciences, University of Nevada at Las Vegas, Las Vegas, Nevada 89154-4004, USA.

Supplementary Figures

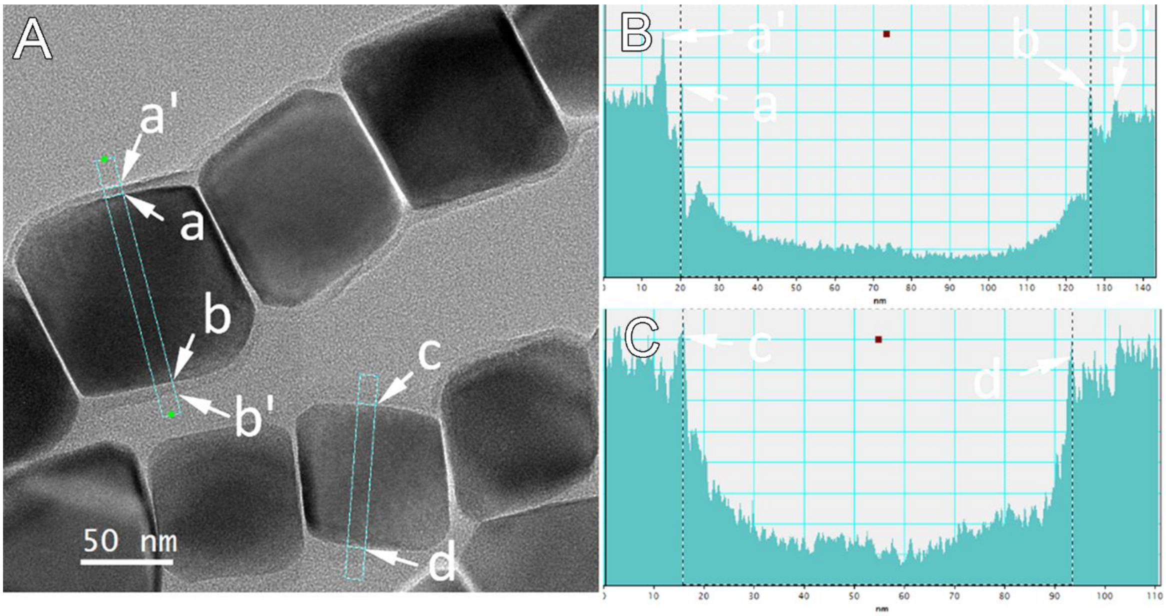

Figure S1. Conventional high resolution transmission electron microscopy (HRTEM) phase contrast image of magnetosome using Fresnel Diffracting contrast. The bright fringe a,a’ b,b’ and c,d, known as the Fresnel fringe, observed at the interface between two materials of different natures or thicknesses, derives from the interference of the waves passing through these two materials, which have undergone a different modification of their phase. Their observation depends on the value of the defocusing of the objective lens (28). Detection of the magnetosome membrane is dependent on the relative thickness of the crystal and the membrane. A) Chain of purified Fe_3O_4 magnetosomes from cultured *Magnetofaba australis* strain IT-1 cells. Regions from where the intensity profiles of the images were extracted. B) Intensity profiles in a thick region in which the Fresnel diffracting contrast a,a’ and b,b’ highlights the presence of the phospholipid bilayer membrane with thickness of 3nm for aa’ and 4nm for b,b’. C) Profile of a thinner Fe_3O_4 crystal, Fresnel diffraction contrast is too low to see the

magnetosome membrane on the image 1 (c,d) and to extract a valuable profile in C of the phospholipid bilayer membrane.

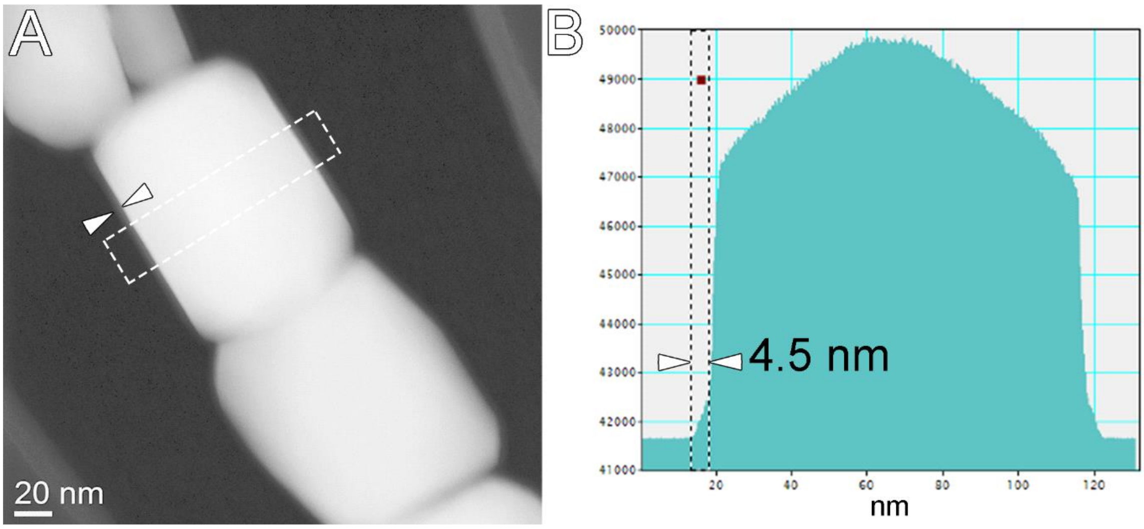

Figure S2. Scanning transmission electron microscopy (STEM) high angle annular dark-field (HAADF) mass contrast image of magnetosome. Contrast of HAADF image is mass thickness dependent. The pixel intensity of the image follows a mass thickness square law (Pennycook, 1989). The presence of the surrounding phospholipid bilayer membrane is highlighted by adjusting the luminosity contrast ratio (A) or by extracting the intensity profile (B) from the HAADF image in A. A) STEM image of a Fe_3O_4 crystal with lateral faces parallel to the electron beam, showing the membrane by modifying the contrast ratio, arrows indicate the external limit of the surrounding membrane. Region indicated by the rectangle was used for the extraction of the intensity profile. B) Corresponding intensity profile (morphological profile) giving the thickness of the membrane (approximately 4.5nm) and the corresponding arrows from A.

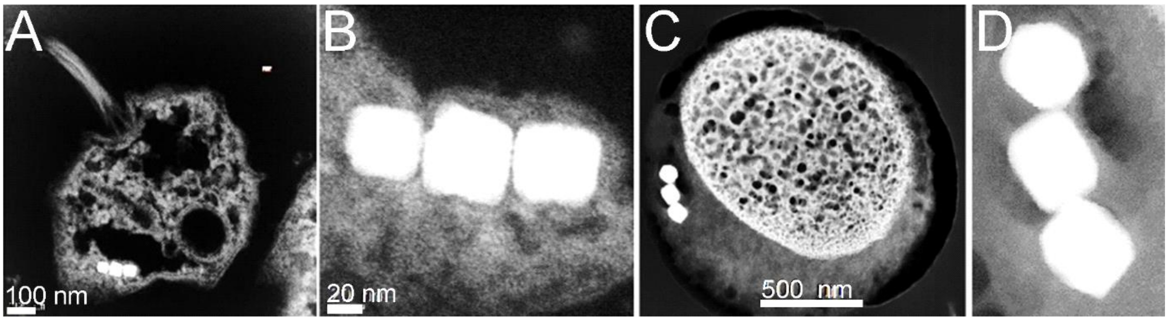

Figure S3. HAADF-STEM of thin-sectioned, cryofixed and freeze substituted cells of various MTB. A) Cell of *Magnetofaba australis* strain IT-1; B) High magnification of cell of *Mf. australis* showing 3 Fe_3O_4 magnetosomes organized in a chain. C) Cell of uncultured freshwater coccus from a pond in Strasbourg, France with Fe_3O_4 magnetosome close to a large porous phosphate granule. D) High magnification HAADF-STEM image of 3 Fe_3O_4 magnetosomes in C organized in a chain. Due to the fact that all these thin-sections were not stained with osmium tetroxide, the magnetosome membranes are not visible.

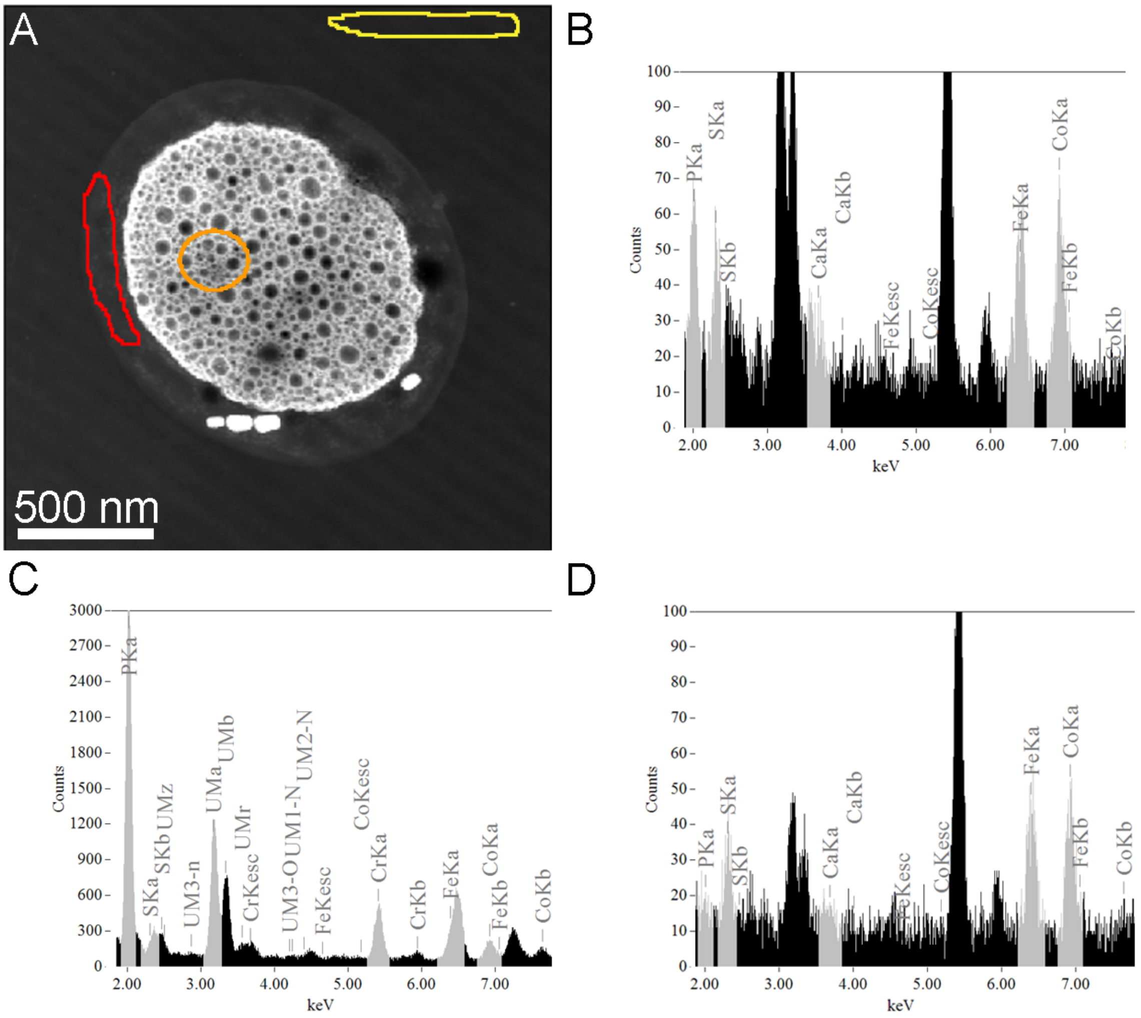

Figure S4. High-sensitivity energy dispersive X-ray (EDS) analyses and elemental mapping of cryofixed thin-section of uncultivated MTB collected from sediments of a pond in Strasbourg, France. The signal of each pixel of the EDS spectrum is contained in a specific mask. The elements C, N, O, Si, P, Cl, Co, Cr and Fe were detected. A) Scanning transmission electron microscopy (STEM) high angle annular dark field (HAADF) image of a thin section of an uncultivated magnetotactic bacterium, showing a large phosphorus-rich granule (likely composed of polyphosphate) surrounded by the cell cytoplasm. The region surrounded by the red line (spectrum B) shows the composition of the cytoplasm containing phosphorus. The region surrounded by an orange circle (spectrum C) shows the composition of the phosphorus-rich granule and the region

surrounded by the yellow line (spectrum D) shows the composition outside the bacterial cell. A high amount of chromium was observed inside and outside the cell, probably originating from the environment. However, phosphorus was only detected inside the granule and in the cytoplasm.

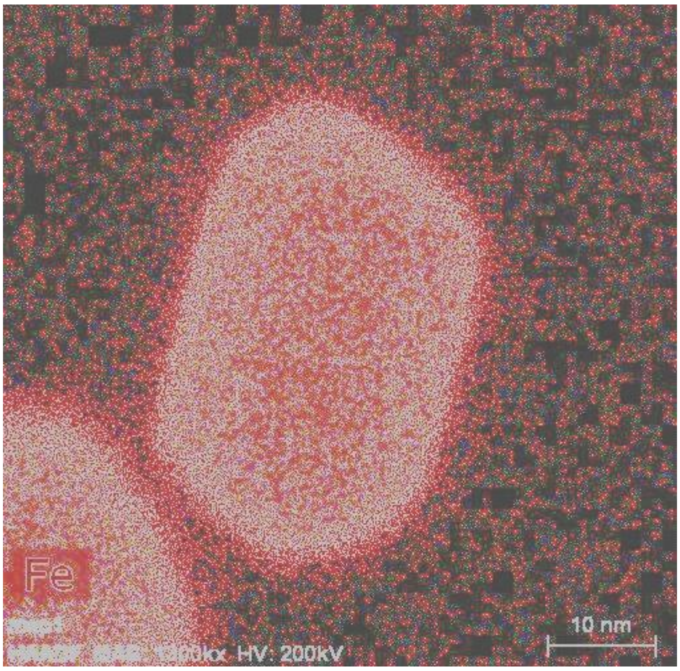

Figure S5. EDS Fe map of a Fe_3O_4 magnetosome in a cell of cultured *Magnetovibrio blakemorei* strain MV-1. Method of edge algorithm was applied to highlight the Fe "corona" surrounding the Fe_3O_4 crystal. EDS elemental mapping was performed using Brucker SDD inside Titan 200 kV beam correction.

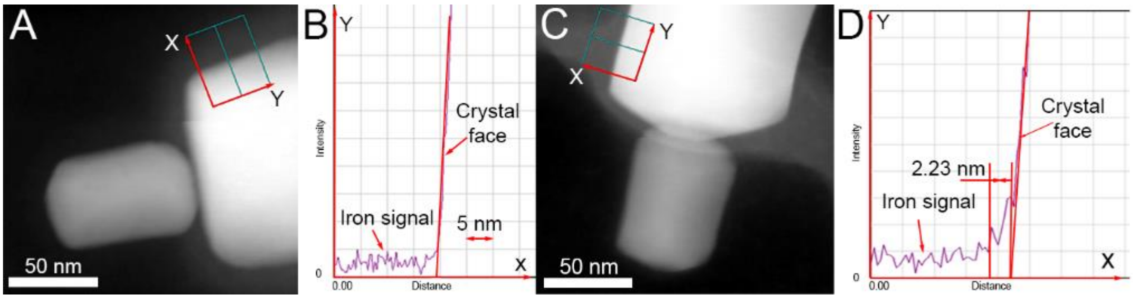

Figure S6. EDX analysis of magnetosomes of two uncultivated magnetotactic cocci from the Mediterranean Sea. A) and C) magnetosomes from cells deposited on the same grid and air dried, with the corresponding analyses shown in B) and D). To obtain a better signal/noise ratio, the signal was added along Y and projected on the X direction. The crystal faces were used as a spatial reference. B) Iron profile signal across the crystal face, no iron is detected outside the crystal. D) Iron profile signal across the crystal face, iron is detected in a region 2.23 nm thick close to the crystal inside the lipid bilayer membrane.

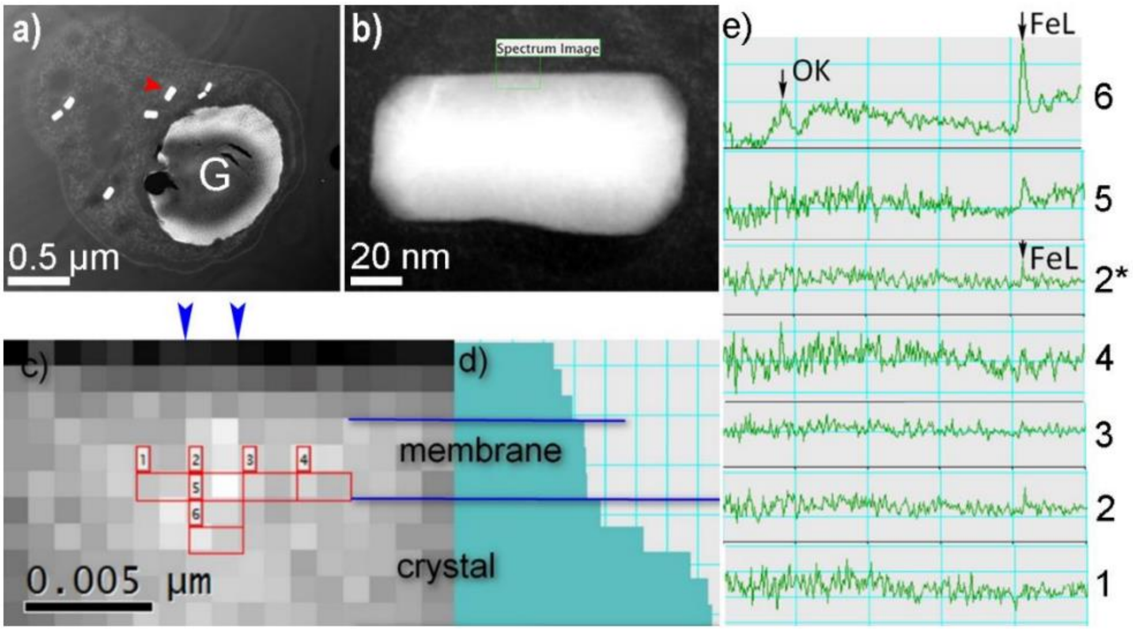

Figure S7. (a) Ultrathin section from rapid-frozen, freeze-substituted uncultivated MTB cells obtained from freshwater sediments in a pond in Strasbourg, France. The red arrow (figure a) indicates a magnetosome used for EELS analysis, shown at high magnification in (b). The blue arrows (figure c) define the width of the two-column pixels used to obtain the morphological profile (figure d). (e) EELS spectra were obtained by the addition of two pixels highlighted by the corresponding red rectangles. From 1 to 4 (beam scanned parallel to the crystal face), EELS analysis shows that the concentration inside the thin layer of membrane is not homogeneous (see spectrum # 2). From spectrum # 2* to spectrum #6, the beam was scanned perpendicular to the crystal face (Dwell time 0.1 s, beam intensity 0.370 nA, camera length 2 cm).
